# Thickness Measurement at High Lift-Off for Underwater Corroded Iron-Steel Structures Using a Magnetic Sensor Probe

**DOI:** 10.3390/s23010380

**Published:** 2022-12-29

**Authors:** Shoya Adachi, Minoru Hayashi, Taisei Kawakami, Yuto Ando, Jin Wang, Kenji Sakai, Toshihiko Kiwa, Toshiyuki Ishikawa, Keiji Tsukada

**Affiliations:** 1 Graduate School of Interdisciplinary Science and Engineering in Health Systems, Okayama University, 3-1-1 Tsushimanaka, Kita-ku, Okayama 700-8530, Japan; 2 Department of Civil, Environmental and Applied System Engineering, Faculty of Environmental and Urban Engineering, Kansai University, 3-3-35 Yamate, Suita 564-8680, Japan

**Keywords:** eddy current testing, high lift-off thickness measurement, magnetic sensor, corrosion, underwater steel structure

## Abstract

Infrastructure facilities that were built approximately half a century ago have rapidly aged. Steel sheet piles, the inspection object in this study, are severely corroded, resulting in cave-in damages at wharfs. To solve such a problem, non-destructive inspection techniques are required. We previously demonstrated plate thickness measurement using extremely low-frequency eddy current testing. However, when the steel sheet piles are located in water, shellfish adhere to their surface, causing a lift-off of several tens of millimeters. Therefore, this large lift-off hinders the thickness measurement owing to fluctuations of magnetic signals. In this study, sensor probes with different coil diameters were prototyped and the optimum size for measuring steel sheet piles at high lift-off was investigated. Using the probes, the magnetic field was applied with a lift-off range from 0 to 80 mm, and the intensity and phase of the detected magnetic field were analyzed. Subsequently, by increasing the probe diameter, a good sensitivity was obtained for the thickness estimation with a lift-off of up to 60 mm. Moreover, these probes were used to measure the thickness of actual steel sheet piles, and measurements were successfully obtained at a high lift-off.

## 1. Introduction

Civil infrastructures that are essential to daily economic activities, such as bridges, tunnels, and aqueducts, have aged, and several of them are approaching their designed service life. This increases the risk of collapse and threatens people’s lives. Among the factors that lead to such accidents, the most destructive is metal corrosion. Metal corrosion is also a consequence of aging, but natural environmental factors, such as salt damage and animal fecal damage, accelerate the corrosion process. Therefore, aging infrastructure facilities require continuous maintenance and monitoring. The development of non-destructive testing (NDT) techniques is desired because they enable efficient inspection of structures without causing damages.

NDT methods are utilized to detect existing and unverified defects without causing any damage to structural materials. Various techniques have been implemented for the inspection depending on the defect type, position, and inspection objects [1]. The most common and fastest NDT method is visual testing (VT) [2]. Although VT is typically performed with the naked eye, binoculars and scopes are used in some cases. Recently, drones and robots have been utilized, providing efficient assessments. However, VT is limited to detection of visible external and surface defects. Magnetic particle testing (MT) with iron particles is used for surface detection, and radiographic testing (RT) with X-rays or gamma-rays is mainly used for detection of internal and subsurface defects, such as cracks in welded joints. Although RT provides excellent recordability of thickness and defects [1,3], it requires more time and considerations for safety. Among various NDT methods, VT and MT are commonly used for underwater detection, such as offshore oil and gas pipeline [4]. However, a more efficient method is required because the number of aging infrastructures continues to increase. For detection of internal defects including thickness measurement and underwater detection, ultrasonic testing (UT) is an easy NDT method that is more commonly used.

Steel sheet piles, the inspection object of this study, are one of the aging infrastructure facilities that have suffered from serious corrosion damage. Steel sheet piles are used in port areas to protect wharfs from erosion, but their corrosion is accelerated due to chlorine ions in seawater and continuous wave hitting. The sand under a wharf flows away from the corrosion hole, and the wharf becomes exposed to seawater, which causes cave-in damage. Thus, efficient inspection methods are required to identify the degree of corrosion in steel sheet piles. For corrosion protection, impressed current cathodic protection systems are typically applied to steel sheet piles [5]. Although this method can effectively reduce corrosion, its lifetime is only approximately 20 years. UT has been conventionally employed for the early inspection of steel sheet piles. However, this method requires the removal of deposits on the surface, such as rust, shellfish, and algae, which is difficult in adverse underwater environments, and the thickness evaluation of UT depends on the diver’s skill. Therefore, the application of UT to corrosion inspection of steel sheet piles in port areas has been limited.

Eddy current testing (ECT) does not require removal of deposits or direct contact of the surface. Although ECT is mainly implemented for surface crack detection [6,7,8,9], it can be applied to thickness measurement as well. For thickness measurement by ECT, pulsed waves [10,11,12] and multi-frequency waves [12,13,14,15] are used, and their various applications have been studied. We previously reported extremely low-frequency ECT (ELECT) with a magnetic sensor probe as a thickness measurement method [16]. In ELECT, a low frequency magnetic field under 1 kHz was analyzed using a magnetic sensor with sufficient sensitivity in the low frequency range; thus, the plate thickness was measured because the relationship between phase and plate thickness was obtained [17]. Using ELECT, the thickness measurement of severely corroded iron-steel structures was successfully achieved with accuracy equivalent to that of UT [18]. Therefore, ELECT is an effective method for thickness measurement of steel sheet piles. However, the deposits on the surface of steel sheet piles cause a distance (lift-off) between the probe and the surface. The lift-off due to large shellfish, such as oysters, can reach several tens of millimeters, remarkably attenuating and causing fluctuations in magnetic signals to be detected. Hence, thickness measurement for steel sheet piles in water is difficult. Various studies have been conducted to reduce the effect of lift-off because it substantially limits the application of ECT [19,20,21,22,23]. Nevertheless, an efficient NDT technique that can reduce the lift-off effect over 10 mm has not been developed.

In this study, three sensor probes of ELECT were prototyped with different diameters. Steel plates with different thicknesses were measured using the sensor probes to examine the correlation between the effect of lift-off and probe size. From the measured results, the effect of coil size on the magnetic field intensity and phase was evaluated by changing the lift-off and thickness, and an optimum coil diameter was investigated for the thickness measurement of steel sheet piles at a high lift-off.

## 2. Materials and Methods

**Figure 1:**
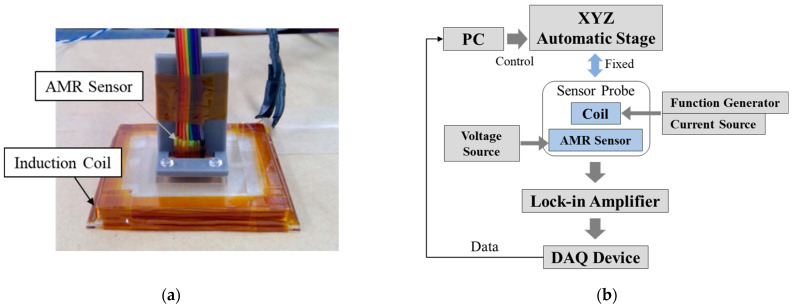
(**a**) Prototype sensor probe, (**b**) Measurement system.

Three sensor probes were prototyped (diameters: 50, 70, 100 mm) to compare and evaluate the detected magnetic field due to coil diameters. Figure 1a shows a photograph of the prototype sensor probe. The sensor probes consist of an anisotropic magneto-resistive (AMR) sensor (Honeywell, HMC1001) and an induction coil. The induction coil was wound with a copper wire (turn number: 10, wire diameter: Φ 0.5 mm), and protected by an insulation tape.

Figure 1b shows the measurement system, which consists of voltage source, current source, function generator, sensor probe, analog-to-digital converter, lock-in amplifier, personal computer (PC), and XYZ stage. The AMR sensor was driven by the 12-V voltage source, and a sine wave current of 200 mA_p-p_ with multiple frequencies (3, 20, 1000 Hz) was applied to the induction coil by the function generator and current source. Steel plates (SM400, 300 × 300 mm^2^) with thicknesses of 3, 5, 7, 9 mm were used as measurement objects. In the measurement of steel plate thickness, the lift-off was changed by 20 mm in the range from 0 to 80 mm by shifting the stage in the Z direction, and the secondary magnetic field excited by eddy currents was detected.

**Figure 2:**
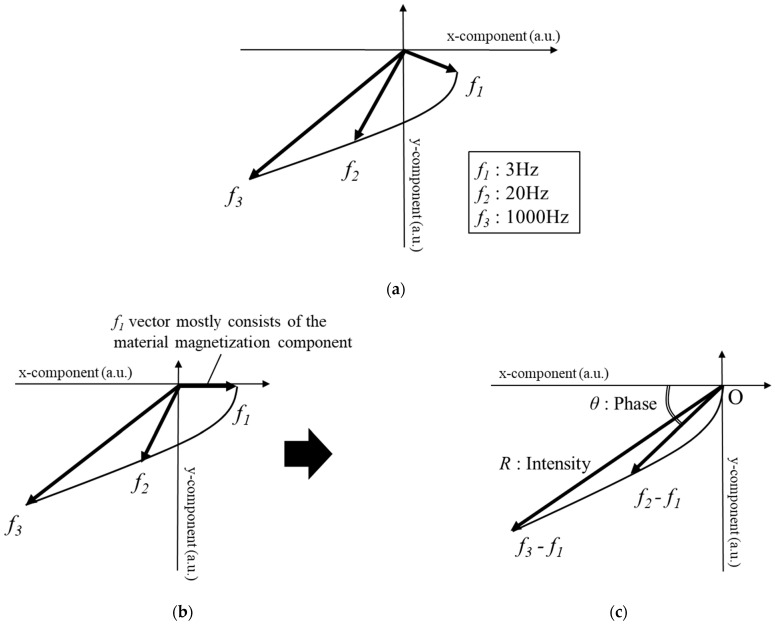
(**a**) Detected magnetic field vectors (**b**) Each vector after the residual magnetization component was subtracted (**c**) True magnetic field vector by eddy current.

Spectroscopy analysis of the magnetic field (SAM) was previously reported as an analysis method of the secondary magnetic field excited by eddy current [16]. According to SAM, the detected magnetic field measured at each frequency can be plotted as magnetic field vectors (Figure 2a). The relationship between the penetration depth *δ* and the frequency *f* by skin effect is crucial to create differential magnetic field vectors and calculate the intensity and phase, given by Equation (1):

(1) $$\delta =\sqrt{\frac{1}{\pi f\sigma \mu }},$$

where *σ* is the conductivity and *μ* is the permeability. As previously mentioned, magnetic fields at 3, 20, 1000 Hz were applied in this study. According to Equation (1), as the magnetic field at a low frequency (20 Hz) can penetrate the steel plate, the magnetic response characteristic, which depends on the plate thickness, can be acquired. By contrast, the magnetic field at a high frequency (1000 Hz) penetrates only near the surface due to the skin effect, and the magnetic response characteristic depends on the plate thickness and lift-off.

The detected magnetic field can be classified into three components: true magnetic field created by eddy current, residual magnetization in the coil, and material magnetization component. The true magnetic field caused by eddy current can be obtained by subtracting the other components. The residual magnetization component can be measured in the air or at a distance from metallic objects, whereas the material magnetization component can be measured by subtracting the magnetic field of the lowest frequency (Figure 2b). In comparison with other frequency magnetic fields, the magnetic field of the lowest frequency is primarily composed of the material magnetization component, and few true magnetic fields are included. Therefore, by subtracting the 3-Hz vector from the other frequency vectors (hereinafter referred to as 20–3 and 1000–3 Hz), the true magnetic field vectors generated by eddy current can be acquired and the intensity and phase are then calculated from the true magnetic field vectors (Figure 2c).

## 3. Simulations of Magnetic Distributions

**Figure 3:**
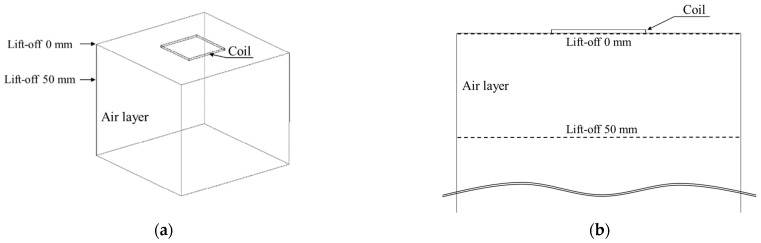
Photograph of the computer-aided wireframe design model (these models show a 100-mm² coil): (**a**) whole image and (**b**) cross-sectional image.

To qualitatively evaluate the difference in the applied magnetic field due to the coil diameters and lift-off, simulations of magnetic distribution by changing the coil diameter and lift-off were performed using the commercially available electromagnetic analysis software JMAG (JSOL Corporation). The simulation models were assembled with an air layer element and a coil element (diameters: 50, 70, 100 mm^2^), whose inside was full of an air layer (Figure 3a,b). The resistance values of each coil were set to 0.5, 0.7, and 0.9 Ω, respectively, and a magnetic field (200 mA_p-p_, 1000 Hz) was applied to the coil. All elements were divided into 1 mm meshes for the finite-element analysis. The air layer was a 300-mm^3^ cube and was cut at the plane of lift-offs 0 and 50 mm after the calculation to obtain the magnetic field distribution diagrams.

**Figure 4:**
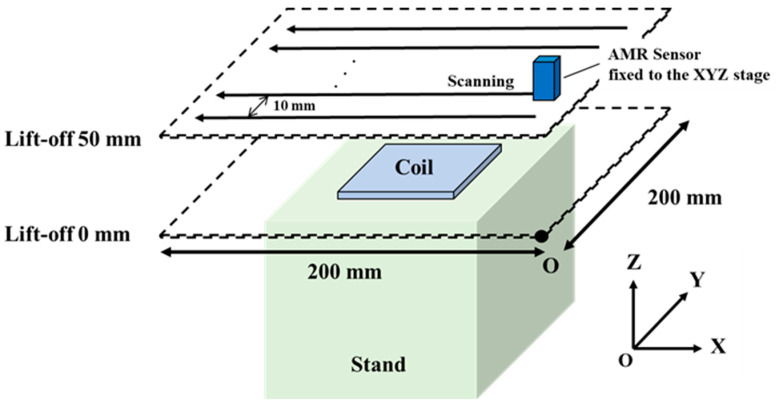
Schematic of magnetic field distribution measurement.

Mapping diagrams of actual magnetic field distributions were obtained with the prototype sensor probes. A magnetic field (200 mA_p-p_, 1000 Hz) was applied to the induction coil, which was fixed on the stand to adjust the lift-off. An AMR sensor was fixed to the XYZ stage and was automatically scanned in the X and Y directions every 10 mm until 200 mm at a speed of 10 m/s (Figure 4). The lift-off was changed by shifting the stage in the Z direction from 0 to 50 mm.

**Figure 5:**
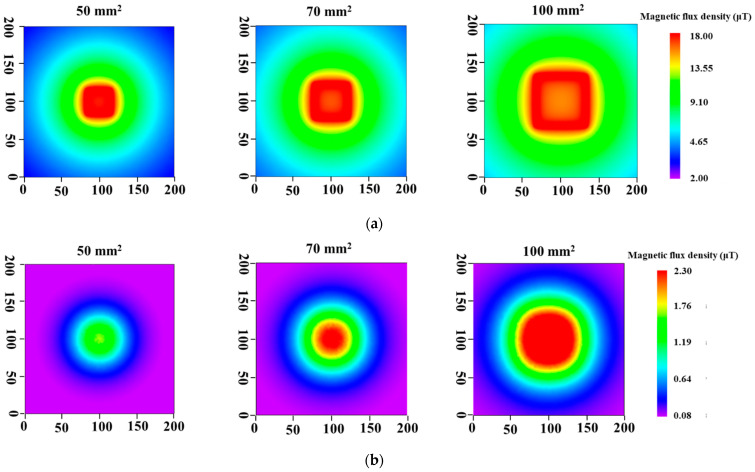
Simulation of magnetic field distributions at lift-offs: (**a**) 0 mm and (**b**) 50 mm.

Figure 5 shows the results of simulations. When the lift-off was 0 mm, intense magnetic fields were observed over a wide range as the coil diameter increased, but the magnetic field in the center part became weak, as shown in Figure 5a. By contrast, the magnetic field distributions at the lift-off of 50 mm showed that the intensity became weak and the range of magnetic field for the distributions became narrow compared with those obtained at the lift-off of 0 mm (Figure 5b). However, the intensity increased as the coil size became larger.

**Figure 6:**
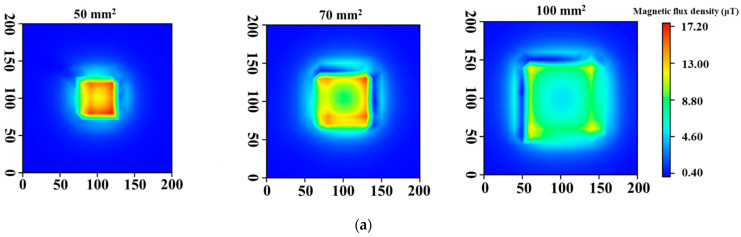

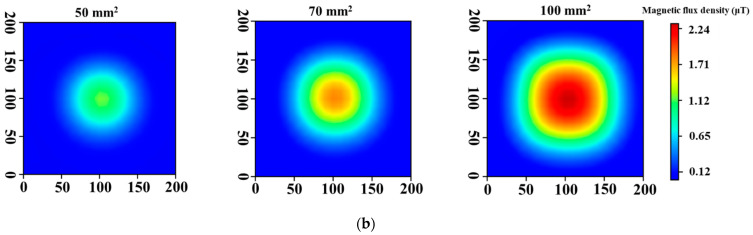
Magnetic field distributions with prototyped probes at lift-offs: (**a**) 0 mm and (**b**) 50 mm.

Figure 6 shows the magnetic field distributions measured with the prototype probes. The magnetic field distributions at the lift-off of 0 mm were not uniform and the intensity near the center part of the coil was low (Figure 6a). These distortions are attributed to the magnetic flux leakage due to the induction coil, whose winding was not ideal. However, the magnetic field became more extensive as the coil diameter increased. This result is in agreement with simulation results. Moreover, the intensity of the magnetic field at the lift-off of 50 mm also increased and became more extensive (Figure 6b). The magnetic field intensity increased as the coil size increased, and this phenomenon was similar to that in the simulation. These results indicate that thickness measurement at high lift-off values can be conducted by increasing the coil diameter, because a sensor probe with large coil can generate a magnetic field sufficiently large for thickness measurement even when the lift-off increases.

## 4. Results and Discussions

### 4.1. Magnetic Field Intensity versus Lift-Off

**Figure 7:**
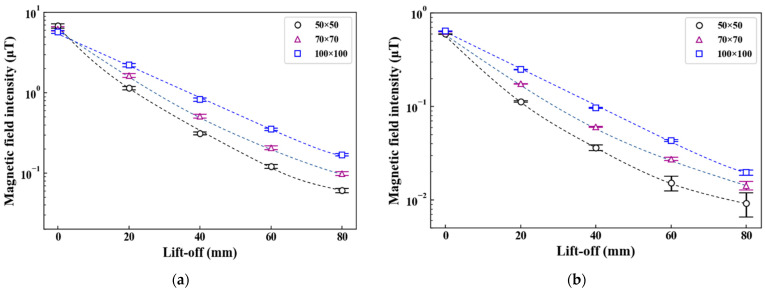
Magnetic field detected at frequencies: (**a**) 1000–3 Hz and (**b**) 20–3 Hz.

The detected magnetic field intensities, which are the subtraction of those obtained at frequencies 20 and 3 Hz (20–3) or 1000 and 3 (1000–3) Hz, were evaluated with the prototype probes (Figure 7). In accordance with the skin effect, the magnetic field at 1000–3 Hz almost depended on the lift-off. Therefore, Figure 7a shows the average values and standard deviations of intensity for each steel plate thickness (3, 5, 7, 9 mm) at the same lift-off. By contrast, the magnetic field at 20–3 Hz depended on the lift-off in addition to the plate thickness. Hence, Figure 7b shows the average values and standard deviations of the intensity at each lift-off for a 7 mm steel plate. To evaluate the attenuation of the detected magnetic field, a simple model of magnetic field by time-varying eddy current density ***j*** was considered. We assumed that an eddy current flows at an arbitrary position r in a steel plate. The retarded vector potential A at point z on the central axis at time *t* is expressed as:

(2) $$A\left(z,t\right)=\frac{\mu_{0}}{4\pi }\int \frac{j\left(r,t-\frac{\left|z-r\right|}{c}\right)}{\left|z-r\right|}dv$$

where *μ*_0_ is the permeability of free space and *c* is the speed of light (All bolds represent vectors). Considering symmetry, the detected magnetic field ***B*** at point z is expressed as:

(3) $$B={\left(\nabla \times A\right)}_{z}$$

From Equations (2) and (3), the magnetic field attenuates in a lift-off-dependent manner. Figure 7a,b show that the intensity became higher as the coil diameter increased, simultaneously showing an attenuation tendency in accordance with Equations (2) and (3). Furthermore, the intensities in Figure 7b at lift-offs 40, 60, and 80 mm were stable when a large coil was used. These results indicate that a stable magnetic field can be detected using a large induction coil, and that this stabilizes the phase with a low frequency of 20–3 Hz.

### 4.2. Phase versus Lift-Off

**Figure 8:**
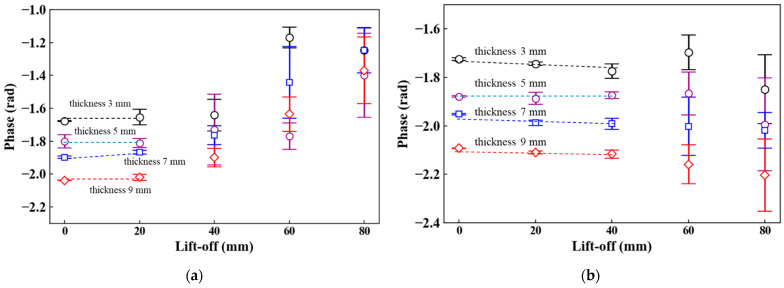

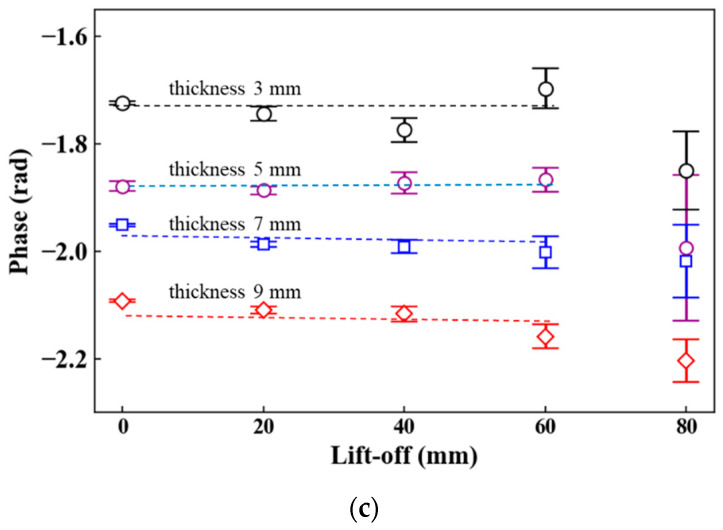
Lift-off dependence of phase (coil area: (**a**) 50 mm^2^, (**b**) 70 mm^2^, (**c**) 100 mm^2^).

The thickness estimation of steel plates can be conducted using calibration curves of phase and thickness. Hence, when the phase values fluctuate due to the lift-off, the thickness estimation becomes difficult. In this section, the phase values at 20–3 Hz were evaluated with the average and standard deviation calculated from five measurements (Figure 8). With the 50-mm^2^ probe, the phase values shifted between 0.1 and 0.2 rad as the lift-off increased (Figure 8a). By contrast, the phase values with the 70-mm^2^ probe were approximately constant in the lift-off range of 0 to 40 mm (Figure 8b). In comparison with the 50 mm^2^ probe, the phase variation was reduced. However, at the lift-offs of 60 and 80 mm, the phase values fluctuated more than 0.1 rad. Figure 8c shows the phase values with the 100-mm^2^ probe. Approximately constant phase values were observed in the lift-off range of 0 to 60 mm. Among the three sensor probes, the phase values were the most stable, and their variations were less than 0.05 rad with the 100-mm^2^ probe. From these results, the 100-mm^2^ probe is preferable to obtain an accurate calibration curve and thickness measurement with the least fluctuations.

### 4.3. Phase versus Plate Thickness

**Figure 9:**
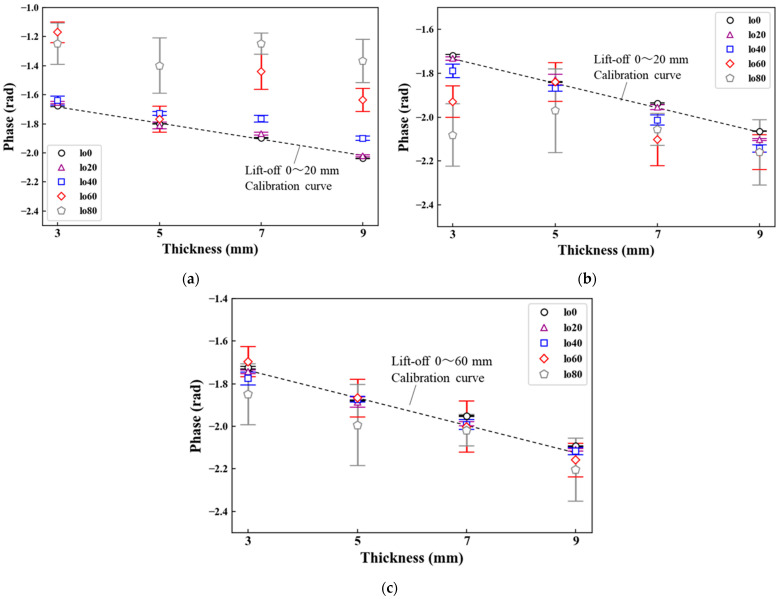
Plate thickness-phase characteristics (coil area: (**a**) 50 mm^2^, (**b**) 70 mm^2^, (**c**) 100 mm^2^).

Figure 9 shows the relationship between the plate thickness and the phase. In general, the phase exponentially decreases with increasing plate thickness and gradually saturates [18]. Fluctuations of the phase due to the lift-off should be reduced in a linear region of the calibration curve to accurately estimate the plate thickness [17]. When the 50-mm^2^ probe was used, a liner correlation between the phase and thickness was expressed by one calibration curve with the lift-off from 0 to 20 mm (Figure 9a). However, in the lift-off range of 40 to 80 mm, obtaining the calibration curve was difficult because the phase values were neither constant nor monotonically decreased. With the 70-mm^2^ probe, the linear correlation between the phase and thickness was observed when the lift-off ranged from 0 to 20 mm as well as in the case of the 50-mm^2^ probe, whereas the value at the lift-off of 20 mm was very stable (Figure 9b). Moreover, the phase values monotonically decreased in the lift-off range of 0 to 40 mm despite a mismatch between the phase values at a lift-off of 40 mm and the calibration curve. These results suggest that increasing the coil diameter makes the calibration curve stable. In fact, when the 100-mm^2^ probe was used, the characteristic in the lift-off range of 0 to 60 mm was expressed by one calibration curve (Figure 9c). Compared with the other two probes, the 100-mm^2^ probe reduced the fluctuations of the phase values, particularly at a lift-off of 40 mm where the variation was less than 0.02 rad with all given thicknesses. Additionally, although the phase values fluctuated at a lift-off 60 mm, the phase monotonically decreased with increasing thickness along with the calibration curve. From the above results, it is evident the thickness measurement at high lift-offs can be performed by increasing the coil diameter. Developing a 100-mm^2^ sensor probe for underwater use should be considered to realize thickness estimation at high lift-offs for actual steel sheet piles.

## 5. Demonstration of Thickness Measurements at High Lift-Off

### 5.1. A Newly Developed Magnetic Sensor Probe and a Portable ELECT Device

**Figure 10:**
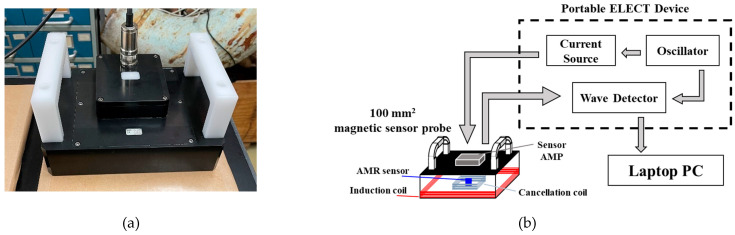
(**a**) 100-mm^2^ sensor probe; (**b**) ELECT measurement system.

The prototype sensor probe of 100 mm^2^ achieved the most sensitive detection of magnetic field at high lift-offs. To validate this result, we developed a 100-mm^2^ magnetic sensor probe for underwater use for measuring an actual corroded steel sheet pile in the port area (Figure 10a). A portable ELECT device was used for the on-site experiment (Figure 10b). The sensor probe was waterproofed and consisted of an AMR sensor, a sensor amplifier, an induction coil (200 turns of copper wire with wire diameter Φ of 0.3 mm), and a cancellation coil (137 turns of copper wire with wire diameter Φ of 0.3 mm). The cancellation coil was wound with copper wire in the reverse direction to the induction coil. These two coils were connected in series to each other. The portable ELECT device consists of a current source, oscillator, and wave detector, and can be connected to the sensor probe with a waterproof cable. By controlling a PC, the applied frequencies and current were set, and measurement data were acquired.

### 5.2. Measurement Conditions

**Figure 11:**
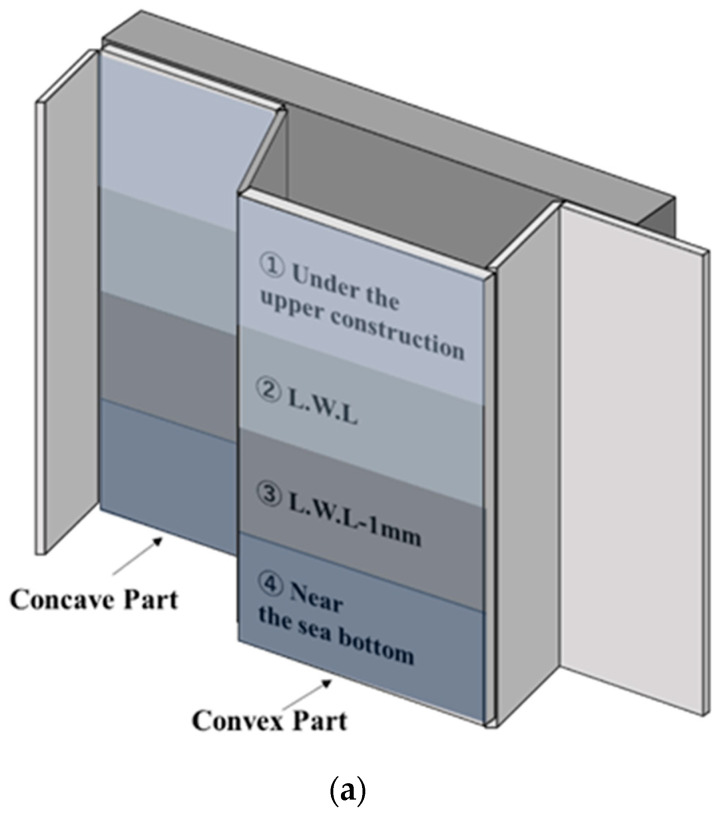

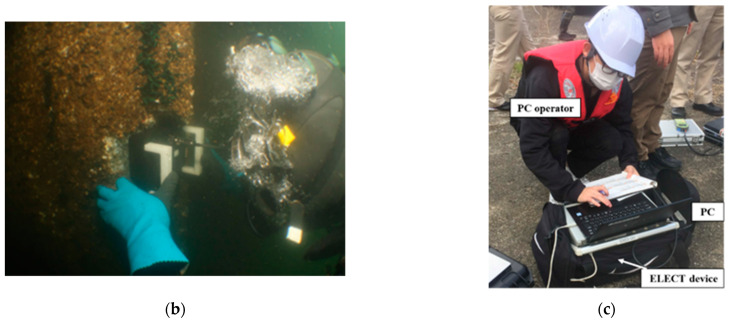
(**a**) Schematic of a steel sheet pile; (**b**) Photographs of the diver measuring a steel sheet pile in water; (**c**) PC operator.

In this demonstration, corroded steel sheet piles installed in the 1970s were measured. The measurement was conducted in concave and convex parts at four depths (Figure 11a). Although the weather was sunny and the sea was calm on the measurement day, the underwater environment was muddy. A diver set up the sensor probe in water and removed the surface deposits (Keren). Simultaneously, workers on the ground operated the PC and communicated with the diver with a wireless device, and the measurement was performed (Figure 11b,c). First, ELECT was applied to the steel sheet pile whose surface deposits were removed. Second, ELECT and UT were applied over Keren.

The calibration curve was obtained from two types of steel plate (SS400, SM400) using the newly developed sensor probe. In the study by Tsukada et al. [17], a linear region of the calibration curve changed depending on a frequency set of the differential magnetic field vectors. Therefore, a frequency set should be adequately selected such that the linear region includes the thickness of the measurement object. As the original thickness of the steel sheet piles was 16.1 mm, the applied current and frequencies were set to 200 mA_p-p_ and 1000–3, 5–3 Hz, respectively [17]. Calibration curves were created for each frequency set. From 1000–3 Hz, a characteristic of intensity and lift-off was obtained, and the lift-off was estimated. In contrast, a characteristic of phase and plate thickness was obtained with 5–3 Hz and the thickness was estimated.

### 5.3. Results of the Demonstration

The measured results of thickness using ELECT and UT are listed in Table 1. The estimated lift-offs using a magnetic field intensity of 1000–3 Hz were 5–20 mm in both concave and convex parts at all depths (Table 1). In the concave part, the estimated thicknesses at high lift-off almost corresponded to the results of ELECT with Keren and the conventional method UT. Errors between these three methods were 0.6 mm at most. In the convex part, the results of ELECT almost corresponded to those of UT, similar to the case of the concave part, with errors of up to 0.3 mm. From these results, thickness measurement at a high lift-off was successfully achieved.

## 6. Conclusions

In this study, the effect of induction coil diameter on the detected magnetic field for various lift-offs was investigated to measure the steel plate thickness at high lift-offs. Simulations of applied magnetic field showed that an intense and extensive magnetic field could be applied more uniformly using a large diameter coil. In fact, a more intense and stable magnetic field was detected using a 100-mm^2^ magnetic sensor probe, despite the increase in the lift-off. As the fluctuations of phase-thickness characteristics were reduced using a large coil, the lift-off limit for thickness measurement was improved from 5 mm to 60 mm. Based on these results, the thickness of corroded steel sheet piles underwater was measured. The thickness measurement at a high lift-off was successfully achieved with accuracy equivalent to that of UT. In this study, the calibration curve was obtained from two types of steel plate. As phase values vary depending on the electromagnetic parameters, such as conductivity and permeability, revealing the relationship between these parameters and the calibration curve is a crucial issue for future research. Regardless of the steel type, ELECT can be successfully used for thickness measurement.

## Figures and Tables

**Table 1:** Comparisons of estimated thickness.

Original Thickness 16.1 mm		Estimated Thickness (mm) The Numbers in Parentheses Show Estimated Lift-Off (mm)			
		① Under the Upper Construction	② L.W.L	③ L.W.L −1 m	④ Near the Sea bottom
ELECT Before Keren	Concave	15.1 (16.3)	14.9 (10.6)	14.0 (7.0)	14.6 (5.2)
	Convex	14.6 (19.3)	14.7 (8.5)	14.6 (6.0)	15.0 (6.7)
ELECT After Keren	Concave	14.9	14.7	14.2	14.3
	Convex	14.4	14.9	14.7	14.8
UT	Concave	14.7	14.5	14.6	14.7
	Convex	14.6	14.6	14.5	14.5

## Data Availability

The data that support the findings of this study are available from the corresponding author, [K.T.], upon reasonable request.
